# Analgesic efficacy of intravenous nefopam after spine surgery: a randomized, double-blind, placebo-controlled trial

**DOI:** 10.12688/f1000research.22909.2

**Published:** 2020-08-26

**Authors:** Jatuporn Eiamcharoenwit, Haruthai Chotisukarat, Kanjana Tainil, Nalinrat Attanath, Phuping Akavipat

**Affiliations:** 1 Department of Anesthesiology, Prasat Neurological Institute, 312, Ratchawithi Road, Thung Phaya Thai, Ratchathewi, Bangkok, 10400, Thailand

**Keywords:** Non-opioid analgesics; Postoperative pain; Morphine consumption

## Abstract

**Background** **:** The incidence of moderate to severe pain is high among patients undergoing spinal surgery. Nefopam can be used as an adjuvant analgesic postoperatively after spine surgery. The study aimed to assess the analgesic efficacy and side effects of nefopam on 24-hour postoperative morphine consumption after spine surgery.

**Methods** **:** The study is a randomized, double-blinded, placebo-controlled trial. A total of 96 patients were randomized into 4 treatment groups, 24 each. In group 1, patients received normal saline before surgical incision and before the end of surgery. In group 2, patients received 30 mg nefopam before surgical incision and normal saline before the end of surgery. In group 3, patients received normal saline before surgical incision and 30 mg of nefopam before the end of surgery. In group 4, patients received 30 mg of nefopam in both timings. Patient-controlled analgesia morphine was used for the postoperative period. Outcomes were to determine 24-hour morphine consumption and incidence of side effects.

**Results** **:** Of 96 patients enrolled, 21 in placebo-placebo, 22 in nefopam-placebo, 22 in placebo-nefopam and 21 in nefopam-nefopam groups completed the study. Analysis of the Kruskal-Wallis test shows no significant difference in 24-hour postoperative morphine consumption between four groups, which were 18 [IQR 13.5-29], 20 [IQR 11-28.3], 17 [IQR 11.5-28.5], 13 [IQR 8.5-18.5] mg., respectively (p = 0.223).  Incidence of side effects, including tachycardia, sedation, sweating and nausea/ vomiting, did not differ.

**Conclusions** **:** Adding perioperative nefopam to opioid analgesic does not improve analgesic efficacy in patients who underwent spine surgery.

**Registration** **:** Thai Clinical Trials Registry ID TCTR20171115001; registered on 15 November 2017.

## Introduction

The incidence of moderate to severe pain after spine surgery is 30–64%, especially in the first 3 days after surgery ^1, 2^. Currently, opioids are primarily considered for postoperative pain control. However, a high dose of opioids may cause side effects such as nausea, vomiting, drowsiness and respiratory depression ^3, 4^. As a result, patients recover slowly after surgery ^1, 5^.

In addition to opioids, adjuvant drugs are also used for postoperatively after spine surgery to reduce the amount and side effects of opioids, including non-steroidal anti-inflammatory, drugs: NSAIDs, gabapentinoids (such as pregabalin gabapentin) and paracetamol ^6– 9^. NSAIDs work by inhibiting the production of prostaglandins both in the central nervous system and peripheral nervous system through the inhibition of cyclooxygenase (COX) isoenzymes. The results of these effects reduce inflammation and pain after surgery. Although NSAIDs have an opioid-sparing effect, there are adverse effects; traditional NSAIDs inhibit the aggregation of platelets and may cause abnormal bleeding during surgery, increasing the risk of bleeding from ulcers in the gastrointestinal tract. In patients receiving COX-2 inhibitors, the risk of thrombosis increases, especially the coronary arteries. Also, elderly patients receiving NSAIDs may exhibit impaired kidney function, potentially leading to acute renal failure ^10^.

Nefopam is a non-opioid analgesic drug used to treat postoperative pain. Mechanism of action is inhibiting the re-uptake of serotonin and norepinephrine ^11^. It also reduces glutamate release via modulating sodium and calcium channels ^12^. In previous studies, multiple timings of systemic nefopam were administered during the perioperative period. Nefopam was administered either before surgical incision, defined as preemptive analgesia ^13– 16^, or at the end of surgery ^17– 23^ for post-operative pain control; however, the correct timing is not known. Therefore, the objectives of this study were to determine the analgesic efficacy and side effects of nefopam that administered before surgical incision, or before the end of the surgery, or both timings compared with placebo on postoperative morphine consumption.

## Methods

### Ethical issues

The study was a randomized, double-blinded, placebo-controlled trial. It was approved by the Institutional Review Board of Prasat Neurological Institute (IRBPNI) [Bangkok] and informed consent was obtained from all patients. The patients enrolled in the study comprised all patients undergoing spine surgery at Prasat Neurological Institute, February 2018 to March 2019.

### Recruitment and allocation

Inclusion criteria were patient with age >18 years, who were undergoing lumbosacral spine surgery under general anesthesia; elective case; not more than three-level spinal surgery; ASA physical status I-III; expected length of operation of 4–6 hours; and no history of nefopam or morphine allergy. Exclusion criteria were: patients with ischemic heart disease or arrhythmia; epilepsy; liver disease; creatinine clearance <30 ml/min; receiving nefopam within 24 hours or five elimination half-lives of nefopam; received strong opioids for more than 2 weeks or received monoamine oxidase inhibitor within 2 weeks before surgery; and who are unable to use a patient-controlled analgesia (PCA) device.

Patients were allocated into four treatment groups by the computer-generated random sequence and their allocation placed in a sealed envelope. A total of 96 patients were randomized into 4 treatment groups: 24 in placebo-placebo, 24 in nefopam-placebo, 24 in placebo-nefopam and 24 in nefopam-nefopam groups. The envelopes were opened only after the enrolled participants by the nurse anesthetists who was not involved in the study. All participants and researchers were blinded to the group allocation. At enrolment, patients were explained on a 0 to 10 numerical rating scale (NRS): 0 corresponds to no pain and 10 to the worst imaginable pain for postoperative pain assessment, and how to use the patient-controlled analgesia device on a day before surgery. Patients received premedication with 7.5 mg of midazolam within 30 – 60 minutes before anesthesia. No patients received NSAIDs, serotonin and norepinephrine reuptake inhibitors, tricyclic antidepressant, gabapentinoid or opioids on the morning of surgery.

### Intervention

When the patients arrived at the operating theatre, each group of patients received two treatment timings: period A, 30 minutes before surgical incision; or period B, 30 minutes before the end of surgery or both timings compared with matching placebo. Nurse anesthetists not involved in the research project opened sealed envelopes containing the allocation to group in the order of patients. Study medications were in identical appearance bottles; 100 ml of transparent, colorless solution, containing either 30 mg of nefopam (Acupan® BIOCODEX) or placebo, which was prepared in sealed envelopes by the nurse anesthetist who was not involved in the study. Study medications were given intravenously within 20 minutes each time. The treatment team and the data collector will not know which group of participants is in the study group. The patients were withdrawn and labels were opened if they exhibited a heart rate >150/min, arrhythmia, development of extreme unexpected events (such as acute ischemic heart disease, pulmonary embolism), and if the patient stopped using the PCA device before 24 hours after surgery.

In the operating theatre, Patients were monitored routinely with an electrocardiogram, noninvasive blood pressure, and pulse oximetry. Additionally, a bispectral index (BIS) monitor was utilized to assess the depth of anesthesia. Pre-oxygenation with high-flow oxygen through a facemask was given for 3 to 5 minutes. Anesthesia was then induced with intravenous propofol (1.5–2 mg/kg), and intravenous cisatracurium (0.15–0.2 mg/kg) was administered to facilitate the endotracheal intubation. Anesthesia was maintained at 1 MAC of desflurane with oxygen and nitrous oxide. Anesthesiologists provided cisatracurium and morphine by adjusting the depth of anesthesia to the Bispectral index (BIS) of 40–60. All patients received local wound infiltration with 20 ml of 0.5% bupivacaine at the end of the operation. At the recovery room, the patients were asked pain scores every 15 minutes using a numerical rating scale. If the pain score is greater than or equal to 4 points, the patients were injected with 2 mg of morphine every 10 minutes until the patients reported pain scores of less than 4. Then, the patients started to use the PCA device in the post-operative period. The PCA devices used in this study were IVAC® PCAM® Syringe Pumps (Alaris, United Kingdom). The protocol PCA setting of morphine 1 mg/ml; no basal rate, bolus dose of 1 mg, lockout interval of 5 minutes, 4-hour limit of 40 mg. If the patients required more than 40 mg of morphine within 4 hours, the cause of pain was reevaluated and neuropathic pain was ruled out by using the Thai-language Neuropathic Pain Diagnostic Questionnaire (Thai DN4) ^24^. Any other analgesics were not permitted during the study period.

### Outcomes

Demographic data, American Society of Anesthesiologist (ASA) Physical status, comorbid disease, average pain score in a 24-hour period before surgery, operation, anesthetic time, duration of surgery, vital signs every 2.5 minutes during nefopam administration, intraoperative and 24 hours postoperative morphine consumption, first time to rescue morphine, pain score during postoperative 24 hours and side effects were recorded.

The primary outcome of the study was to determine the analgesic efficacy. The primary efficacy was defined as the cumulative morphine dose received 24 hours postoperatively by PCA during each time of nefopam administration. Secondary outcome comparisons were 24-hour postoperative pain score, and incidence of side effects such as tachycardia, sedation, sweating and nausea/vomiting. Sedation was defined as a Pasero Opioid-induced Sedation Scale (POSS) score that was greater than or equal to 3 ^25^. Clinically important postoperative nausea and vomiting (PONV) were defined as PONV intensity scale of ≥75 ^26^.

### Sample size calculation and statistical analysis

Previous studies have shown that when nefopam was given before the end of surgery, postoperatively 24-hour morphine consumption was 21.2 (15.3) mg. Morphine dose received 24 hours postoperatively in control group was 27.3 (19.2) mg ^17^. The sample size was determined from total postoperatively 24-hour morphine consumption. Neither the mean of 24-hour morphine consumption in the group receiving nefopam before surgical incision nor the group receiving nefopam before surgical incision plus at the end of surgery compared with placebo was reported in these previous studies. Therefore, this sample size estimated the level of reduction in 24-hour morphine consumption in both groups. The sample size gives the trial a power of 80%, sets a two-tailed α at 0.05 in means characterized by a variance of means of 11.792, assuming that the common standard deviation is 9.12. The calculation resulted in 21 patients per group. To compensate for 10% attrition rate, we included 24 patients per group. The total sample size is 96 patients. Criteria for interim analysis and early termination of the study were as follows: 1) a heart rate of more than 150 beats per minute; 2) arrhythmias; and 3) patients developed extreme unexpected events such as acute ischemic heart disease, pulmonary embolism. Analysis of the analgesic efficacy measures was performed by the Kruskal-Wallis test. Descriptive statistical analyses were performed and expressed in median (IQR) for continuous variables and number (percent) for categorical variables as appropriate. The software program SPSS version 16 was used. Safety data analysis was analyzed by a statistical chi-square test. Statistically significance was considered if p-value < 0.05

## Results

### Patient eligibility and background

**Figure 1.**
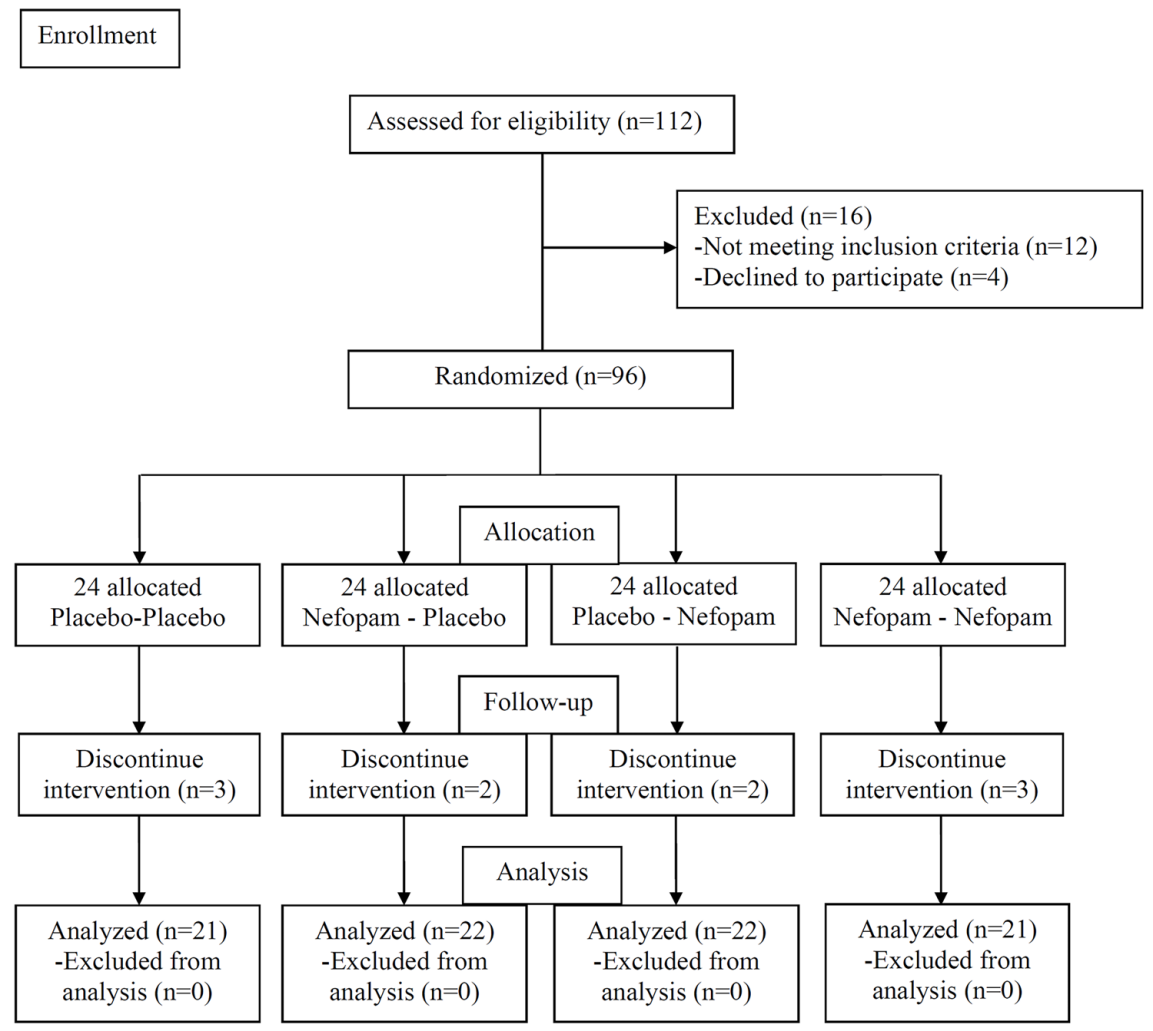
Study flow diagram.

A total of 112 patients were eligible; 12 patients did not meet inclusion criteria and 4 patients declined to participate. As such, 96 patients were randomly assigned to the four groups: 21 in placebo-placebo, 22 in nefopam-placebo, 22 in placebo-nefopam and 21 in nefopam-nefopam groups. There were three patients in the placebo-placebo group, two patients in nefopam-placebo group, two patients in placebo-nefopam group, and three patients in nefopam-nefopam group were excluded from the analysis. Because 10 patients were unable to use patient-controlled analgesia device postoperatively. The total number of patients assessed was 86 ( Figure 1).

Demographic characteristics of patients, preoperative pain score, number of surgical levels, surgery time and anesthetic time are shown in Table 1. The four groups are comparable in age, sex, body mass index, ASA Classification, type of operation, number of levels of spinal surgery, surgery time and anesthetic time. The demographic characteristics and baseline clinical characteristics of patients are similar.

**Table 1.** Demographic characteristics of patients ( *n* = 86). Data given as *n* (%) or median [IQR].

	Group				
Variables	Placebo-Placebo ( *n* = 21)	Nefopam-Placebo ( *n* = 22)	Placebo-Nefopam ( *n* = 22)	Nefopam-Nefopam ( *n* = 21)	p-value
Age, year	67 [52.5-73.5]	57.5 [53.8-63.5]	55.5 [49.8-65.3]	59 [54.5-68.0]	0.082
Female	13 (61.9)	8 (36.4)	14 (63.6)	12 (57.1)	0.242
Body mass index, kg/m ^2^	25.2 [22.5-32.1]	26.3 [23.5-27.3]	27.1 [22.6-29.4]	26.12 [22.4-28.4]	0.984
ASA Classification					0.118
I, II	0, 12 (57.1)	4 (18.2), 16 (72.7)	2 (9.1), 15 (68.2)	2 (9.5), 12 (57.1)	
III	9 (42.9)	2 (9.1)	5 (22.7)	7 (33.3)	
Type of Operation					0.93
TLIF	3 (14.3)	0	0	2 (9.5)	
TPS	17 (81)	22 (100)	21 (95.5)	19 (90.5)	
Other	1 (4.8)	0	1 (4.5)	0	
Number of levels					0.447
1, 2 surgical level	5 (23.8), 7(33.3)	6 (27.3), 10(45.5)	6 (27.3), 6 (27.3)	8 (38.1), 3 (14.3)	
3 surgical levels	9 (42.9)	6 (27.3)	10 (45.5)	10 (47.6)	
Preoperative pain score	3.0 [2-5]	3.0 [1.3-4.8]	3.0 [0-6.0]	4.0 [2.3-5.0]	0.844
Surgery time (min)	264 [233-294]	255 [208-295]	245 [220-280]	247 [226-321]	0.490
Anesthetic time (min)	327 [288-372]	300 [255-352]	306 [289-350]	299 [271-348]	0.836
Intraoperative morphine (mg)	12 [10-15]	12.5 [10-15]	14 [11.5-15]	13 [10-14]	0.498

ASA Classification: American Society of Anesthesiologists Physical Status Classification System; TLIF: transforaminal lumbar interbody fusion; TPS: transpedicular screw.

The postoperative 24-hour morphine consumption (median [IQR]) for the placebo-placebo, nefopam-placebo, placebo-nefopam and nefopam-nefopam groups were not different, at 18 [13.5–29], 20 [11–28.3], 17 [11.5–28.5], 13 [8.5–18.5] mg, respectively (p = 0.223). Time to first dose of morphine were not different, at 30 [7.5–85], 16.5 [10–41], 30 [12.5–67.5], 30 [12.5–91.5] min, respectively (p = 0.710). Pain score and total intraoperative morphine for four treatment groups were not different ( Table 2). All raw data are available from Figshare ^27^.

**Table 2.** Postoperative pain score and postoperative morphine consumption.

Variables *n* (%) or median [IQR]	Placebo-Placebo ( *n* = 21)	Nefopam-Placebo ( *n* = 22)	Placebo-Nefopam ( *n* = 22)	Nefopam-Nefopam ( *n* = 21)	p-value
Pain score					
0 hr	1 [0-5]	4 [0-5]	1 [0-5]	3 [0-5]	0.989
4 hr	5.5 [3-7.8]	5 [4-6.5]	5 [4-8]	6 [5-7]	0.856
8 hr	5 [3-6]	5 [4-6]	4 [3.8-6]	5 [4-6.5]	0.686
12 hr	5 [4.5-6]	5 [3-5.5]	5 [3-7]	5 [3-6]	0.732
16 hr	5 [3.5-5.5]	4 [3-5]	4.5 [3-6.5]	5 [3-6]	0.469
20 hr	4 [3-5]	3.5 [3-5]	3.5 [3-5]	3 [3-5]	0.963
24 hr	4 [3-4.5]	3 [2-4.25]	3.5 [3-5]	4 [3-5]	0.647
Morphine bolus (mg)	0	0 [0-0.5]	0	0 [0-2]	0.236
Morphine consumption (mg)					
0 - 4 hr	3 [1-11]	5 [4-11]	5 [2-6.5]	3 [2-6.5]	0.623
0 - 8 hr	7 [4-16]	9 [6.75-12.3]	9 [3-9.5]	6 [4.5-10]	0.617
0 - 12 hr	11 [6-21.5]	12.5 [9-15.5]	10 [5-15]	9 [6-11.5]	0.361
0 - 16 hr	14 [8-26]	15.5 [11-19.5]	15 [6.5-21.5]	10 [7-14.5]	0.297
0 - 20 hr	16 [12-28]	19 [11-24.8]	17 [9-25]	12 [7.5-16]	0.259
0 - 24 hr	18 [13.5-29]	20 [11-28.3]	17 [11.5-28.5]	13 [8.5-18.5]	0.223

**Figure 2.**
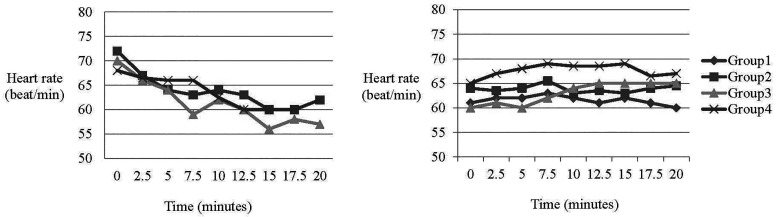
Heart rate in each group (median) before surgical incision (left) and at the end of surgery (right). Group 1: Placebo - Placebo; Group 2: Nefopam - Placebo; Group 3: Placebo - Nefopam; Group 4: Nefopam - Nefopam. There was no statistically significant difference in heart rate between groups.

The incidence of sedation, nausea and vomiting, sweating and intraoperative arrhythmia did not differ significantly between the groups ( Table 3). No patients in four groups developed tachycardia. There was no statistically significant difference in heart rate between the four groups ( Figure 2). No patients discontinued the treatment due to adverse events.

**Table 3.** Side effects.

Variables *number of* *occurrences (%)*	Placebo-Placebo *n* = 21	Nefopam-Placebo *n* = 22	Placebo-Nefopam *n* = 22	Nefopam-Nefopam *n* = 21	p-value
Sedation					
0 – 8 hr	0	3 (13.6)	3 (13.6)	3 (14.3)	0.503
8 – 16 hr	0	1 (4.5)	1 (4.5)	0	0.868
16 – 24 hr	0	0	0	0	-
Nausea and vomiting					
0 – 8 hr	0	1 (4.5)	8 (36.4)	9 (42.9)	0.249
8 – 16 hr	2 (9.5)	1 (4.5)	3 (13.6)	0	0.507
16 – 24 hr	0	1 (4.5)	2 (9.1)	1 (4.8)	1.000
Sweating					
0 – 8 hr	0	1 (4.5)	2 (9.1)	1 (4.8)	1.000
8 – 16 hr	1 (4.8)	1 (4.5)	2 (9.1)	0	0.801
16 – 24 hr	2 (9.5)	0	2 (9.1)	0	0.142
Arrhythmia – Period A	1 (4.8)	2 (9.6)	5 (23)	0	0.801
Arrhythmia – Period B	0	0	0	0	-
At least one occurrence, *n (%)*	1 (4.8)	2 (9.1)	2 (9.1)	3 (14.3)	0.763

Period A: before surgical incision; Period B: before the end of surgery.

## Discussion

The main results of this study showed no difference in 24 hours postoperatively morphine consumption between nefopam group and placebo. Additionally, the analgesic efficacy of nefopam that administered before surgical incision, or before the end of surgery, or both timings compared with placebo were similar. There were no significant differences in early or late side effects between the four groups. The result of this study was similar to previous studies. Merle *et al.* ^19^ and Remérand *et al.* ^20^ reported that nefopam was given at the end of surgery and continuous infusion for 24–48 hours postoperatively, did not reduce morphine consumption. Similarly, Richebé *et al.* ^13^ showed that nefopam given in three periods—at the induction of anesthesia, at the end of surgery and continuous infusion 48 hours postoperatively—did not significant differences in 48 hours morphine consumption. Additionally, Cuvillon *et al.* showed that continuous intravenous 120 mg nefopam and placebo effects were not different at 48 hours after major abdominal surgery ^23^. These previous studies reported the efficacy of nefopam did not significantly differ to placebo, which might be because most of the previous studies studied the usual dose of nefopam 20 mg per dose by intravenous injection. Although intravenous administration of nefopam was given in a single dose of 20 mg, this gave an analgesic efficacy equivalent to 6–12 mg morphine ^17, 28^. On the other hand, many previous studies reported that nefopam had analgesic efficacy to reduce the 24-hours postoperative morphine consumption by up to 19.2–51% ^17, 18^. However, those studies studied patients who underwent minor or moderate surgery, such as laparoscopic or breast surgery, which caused mild to moderate post-operative pain (except for two studies that assessed hip arthroplasty and hepatic resection) ^17, 18^. The recommended dose of nefopam is 20 mg per dose by the manufacturer. The maximum dose of nefopam is no more than 120 mg per day. This study studied in patients who underwent spinal surgery that could cause moderate to severe pain. So, we used the dose of nefopam 30 mg per dose by intravenous injection which the dose injected could be close to the median effective dose (ED50) of studies by Delage *et al.* and Beloeil *et al.* (28 and 27.3 mg), respectively ^29, 30^. The side effects of the ED50 doses were not different from the control group. Another study reported that the ED50 of nefopam for postoperative analgesia in patients who have undergone laparoscopic cholecystectomy was 62.1 mg (95% CI, 52.9–72.9 mg). However, there were 27.6% and 20.7% of the patient developed pain upon injection and phlebitis, respectively ^31^. Nevertheless, the present study assessed a 30-mg dose of nefopam; the main result showed no difference in 24 hours postoperatively morphine consumption between nefopam group and placebo. This study reported negative outcomes, that may be a significant change in research result if 1) extension to the study duration of postoperative nefopam, 2) titration of nefopam dosage achieves an optimal dose and 3) the study design is conducted in patients undergoing minor to moderate surgery.

Our study had some limitations. Firstly, we studied a single dose of nefopam; no continuous intravenous infusion or around-the-clock dosing. However, the half-life of a single dose of nefopam administered intravenously is 3–5 hours, so the analgesic effect of one or two- doses of nefopam did not extend to 24 hours postoperatively. Secondly, almost all patients only experienced preoperative mild to moderate pain. We suggested that perioperative nefopam administration has little analgesic effect, especially when given without opioids. The recommendation for clinical usage is used in combination or adjuvant therapy with the other types of analgesics or multimodal analgesia approach.

## Conclusion

Adding perioperative nefopam to opioid analgesic does not improve analgesic efficacy in patients who underwent spine surgery.

## Data availability

### Underlying data

Figshare: Raw Data: Analgesic Efficacy of Intravenous Nefopam after Spine Surgery: A Randomized, Double-Blind, Placebo-Controlled Trial. https://doi.org/10.6084/m9.figshare.12029256. ^27^

This project contains the individual-level data for each participant.

Figshare: Information of abbreviation data set Title: Analgesic Efficacy of Intravenous Nefopam after Spine Surgery: A Randomized, Double-Blind, Placebo-Controlled Trial Untitled Item. https://doi.org/10.6084/m9.figshare.12090753.v1 ^32^.

This project contains definitions used in the above dataset ^27^.

### Reporting guidelines

Figshare: CONSORT checklist for ‘Analgesic efficacy of intravenous nefopam after spine surgery: a randomized, double-blind, placebo-controlled trial’. https://doi.org/10.6084/m9.figshare.12033693.v4 ^33^.

Data are available under the terms of the Creative Commons Attribution 4.0 International license (CC-BY 4.0).
