# Supplementary material for: Analgesic efficacy of intravenous nefopam after spine surgery: a randomized, double-blind, placebo-controlled trial
Source: F1000Res. 2020 Aug 26;9:516. Originally published 2020 Jun 4. [Version 2] doi: 10.12688/f1000research.22909.2 (PMC7477643; doi:10.12688/f1000research.22909.2)
Supplement: Peer review history

# Peer review: Reviewer response for version 2

**DOI:** 10.5256/f1000research.28724.r70698

**Published:** 2020-09-07

**Authors:** Soe Wunna Htay

**Affiliations:** 1 Defence Services General Hospital, Yangon, Myanmar

This study is a randomized, double-blinded, placebo-controlled trail that used nefopam 30 mg perioperative period for assessing its efficacy in spine surgery. The study reported no morphine consumption reduction in the first 24 hours after spine surgery in the tested groups: placebo-placebo, preincisional nefopam-placebo, placebo-end surgery nefopam, preincisional and end surgery nefopam injfections. The question I want to know is how much the amount of morphine used during the intraoperative period for adjusting the depth of anesthesia to the Bispectral Index (BIS) and should mention first during intervention.

Is the work clearly and accurately presented and does it cite the current literature?

Yes

If applicable, is the statistical analysis and its interpretation appropriate?

Yes

Are all the source data underlying the results available to ensure full reproducibility?

Yes

Is the study design appropriate and is the work technically sound?

Yes

Are the conclusions drawn adequately supported by the results?

Yes

Are sufficient details of methods and analysis provided to allow replication by others?

Yes

Reviewer Expertise:

neuroanaesthesia

I confirm that I have read this submission and believe that I have an appropriate level of expertise to confirm that it is of an acceptable scientific standard.

# Response

**Published:** 2020-09-07

**Authors:** Jatuporn Eiamcharoenwit

**Affiliations:** Department of Anesthesiology, Prasat Neurological Institute, Thailand

Dear Dr. Soe Wunna Htay,

Thank you for your review and comment. Intraoperative morphine (mg) was showed in Table 1. We did not mention the amount of morphine used during the intraoperative period for adjusting the depth of anesthesia. Morphine dosage was at the discretion of anesthesiologists by adjusting the depth of anesthesia to the Bispectral Index (BIS) of 40–60.                                                                                                          

Regards,

Jatuporn Eiamcharoenwit

# Peer review: Reviewer response for version 2

**DOI:** 10.5256/f1000research.28724.r70304

**Published:** 2020-09-03

**Authors:** Rattaphol Seangrung

**Affiliations:** 1 Department of Anesthesiology, Faculty of Medicine Ramathibodi Hospital, Mahidol University, Bangkok, Thailand

No further comments.

Is the work clearly and accurately presented and does it cite the current literature?

Yes

If applicable, is the statistical analysis and its interpretation appropriate?

Yes

Are all the source data underlying the results available to ensure full reproducibility?

Yes

Is the study design appropriate and is the work technically sound?

Yes

Are the conclusions drawn adequately supported by the results?

Yes

Are sufficient details of methods and analysis provided to allow replication by others?

Yes

Reviewer Expertise:

pain management

I confirm that I have read this submission and believe that I have an appropriate level of expertise to confirm that it is of an acceptable scientific standard.

# Peer review: Reviewer response for version 1

**DOI:** 10.5256/f1000research.25291.r65113

**Published:** 2020-07-30

**Authors:** Francis Remérand

**Affiliations:** 1 Anaesthesiology and Critical Care Department, CHRU de Tours, Tours, France; 2 University of Tours, Inserm U1253, Tours, France

This study reports no morphine consumption reduction in the 24 first hours after spine surgery in the 4 tested groups: placebo-placebo, single preincisional nefopam bolus – placebo, placebo-end surgery single nefopam bolus, preincisional and end surgery nefopam injections.

 Despite a well design study (randomized monocentric double blind controlled study), major points have to be clarified, before interpreting the results.

The sample size calculation is unclear: 

- In the methods chapter, it is calculated using a “percentage of reduction" of morphine consumption during the first 24 postoperative hours. The mean consumption to calculate the sample size is not given. By using the shared data of this study, the overall morphine consumption (N=96) is 22 mg +/- 20 (mean +/- standard deviation). With a power of 80%, an alpha at 0.05, 99 patients per group are required to detect the chosen 35% reduction in a two tailed test ( https://biostatgv.sentiweb.fr), while the authors' calculation resulted in 21 patients per group. This study seems therefore largely underpowered.

The perioperative analgesic use should be more accurately described:

- Was intraoperative analgesia obtained using morphine? (Methods, paragraph 6). If yes, the total dose should be mentioned in Table 2 (I suppose the 10-15mg intraoperative morphine mentioned in table 2 is for analgesia, not for a 6 hours anesthesia). Morphine was injected before the PACU in some patients (table 2 and data file): can you describe how the physician decided to inject or not morphine at the end of the surgery?
- Methods, end of paragraph 4: no patient received preoperative gabapentinoid or opiod or NSAID. In the shared data file, 39/96 patients had gabapentinoid, 11 had NSAID and several had tramadol. Please explain these contradictory data.

Data analysis:

- Results, first paragraph: 10 of 96 patients were excluded because operative time < 4 hours - Why did you choose such a limit? This is quite in opposition with the “intention to treat analysis” mentioned in the last paragraph of methods. Please explain why we find 39 patients (and not 10) with an operative time < 240 min in the shared data file. Please clearly identify in the shared file which patients have been excluded.
- How were the lacking data seen in the shared data file managed? (“NA”).

Results:

- Please replace in the third paragraph “similar” by “not different” (2 occurrences).
- Table 3: are we dealing with nb of patients, nb of side effect occurrence? Can you mention cumulative data for patients (nb of patients having at least one occurrence of the side effect during the first 24 hours)?
- I don’t understand Figure 2: can you explain what each “time 0” is?
- The detailed comments on the discussion have to be performed after the above points have been clarified. **The global comment on discussion is that the main limitation of this study is it is largely underpowered**. So conclusions about negative results have to be very cautious (more than in the present version of the paper). Nevertheless, these data have to be published, since they are fitted for a  potential future meta-analysis.

Is the work clearly and accurately presented and does it cite the current literature?

Yes

If applicable, is the statistical analysis and its interpretation appropriate?

No

Are all the source data underlying the results available to ensure full reproducibility?

Partly

Is the study design appropriate and is the work technically sound?

Partly

Are the conclusions drawn adequately supported by the results?

Partly

Are sufficient details of methods and analysis provided to allow replication by others?

Partly

Reviewer Expertise:

anesthesiology and critical care

I confirm that I have read this submission and believe that I have an appropriate level of expertise to confirm that it is of an acceptable scientific standard, however I have significant reservations, as outlined above.

# Response

**Published:** 2020-08-13

**Authors:** Jatuporn Eiamcharoenwit

**Affiliations:** Department of Anesthesiology, Prasat Neurological Institute, Thailand

Dear Dr. Francis Remérand,

Thank you for giving a chance to improve our research article. Hopefully, you would appreciate our revised version.

                                                                                                            

Regards,

Jatuporn Eiamcharoenwit

                                                                                    

**Response to reviewer:**

This study reports no morphine consumption reduction in the 24 first hours after spine surgery in the 4 tested groups: placebo-placebo, single preincisional nefopam bolus – placebo, placebo-end surgery single nefopam bolus, preincisional and end surgery nefopam injections.

Despite a well designed study (randomized monocentric double-blind controlled study), major points have to be clarified, before interpreting the results.

**Comments:**

**The sample size calculation is unclear:**

In the methods chapter, it is calculated using a “percentage of reduction" of morphine consumption during the first 24 postoperative hours. The mean consumption to calculate the sample size is not given. By using the shared data of this study, the overall morphine consumption (N=96) is 22 mg +/- 20 (mean +/- standard deviation). With a power of 80%, an alpha at 0.05, 99 patients per group are required to detect the chosen 35% reduction in a two tailed test (https://biostatgv.sentiweb.fr), while the authors' calculation resulted in 21 patients per group. This study seems therefore largely underpowered.

Response: We changed the primary efficacy to “the primary efficacy was defined as the cumulative morphine dose received 24 hours postoperatively by Patient Controlled Analgesia (PCA) during each time of nefopam administration” (Outcome, paragraph 2).

           

**Response:** According to previous study, PCA-administered morphine over 24 hours was significantly less for the nefopam group than control group 21.2 (15.3) and 27.3 (19.2) mg respectively; P=0.02). In nefopam group, patients received every 4-hour i.v. nefopam 20 mg. The first dose was infused in the operating room at the onset of deep-wound closure ^1^.   

Reference  

1. Du Manoir B, Aubrun F, Langlois M, et al.: Randomized Prospective study of the Analgesic effect of nefopam after orthopaedic surgery. Br J Anaesth. 2003; 91:836-41.

 

We reanalyzed the sample size calculation. We will calculate sample size using One Way Analysis of Variance (ANOVA).

**Table 1:** The cumulative morphine dose received 24 hours postoperatively in 4 group.

Groups                          Cumulative morphine dose received 24 hours postoperatively, mg (SD)

Placebo - Placebo          27.3 (19.2)  using previous data ^1^

Nefopam - Placebo        24                estimated from ascending

Placebo - Nefopam        21.2 (15.3)  using previous data ^1^

Nefopam - Nefopam      18                estimated from descending

Variance of means, V    11.792

**Table 2**: Power and Sample Size for One Way Analysis of Variance (ANOVA)

Test Significance Level, α                 0.05

Number of Groups, G                          4

Variance of Means, V                     11.792

Common Standard Deviation, σ      9.120

Effect Size, Δ² = V/σ²                       0.142

Power (%)                                          80

Sample Size per Group, n                  21

Previous studies have shown that when nefopam was given before the end of surgery, postoperatively 24-hour morphine consumption was 21.2 (15.3). When the sample size in each of the 4 groups is 21, a one-way analysis of variance will have 80% power to detect at the 5% level a difference in means characterized by a variance of means, V, of 11.792, assuming that the common standard deviation is 9.12.

**Response:** We revised information for the sample size calculation and statistical analysis chapter, paragraph 1. “Previous studies have shown that when nefopam was given before the end of surgery, postoperatively 24-hour morphine consumption was 21.2 (15.3) mg. Morphine dose received 24 hours postoperatively in control group was 27.3 (19.2) mg ^1^. The sample size was determined from total postoperatively 24-hour morphine consumption. Neither the mean of 24-hour morphine consumption in the group receiving nefopam before surgical incision nor the group receiving nefopam before surgical incision plus at the end of surgery compared with placebo was reported in these previous studies. Therefore, this sample size estimated the level of reduction in 24-hour morphine consumption in both groups. The sample size gives the trial a power of 80%, sets a two-tailed α at 0.05 in means characterized by a variance of means of 11.792, assuming that the common standard deviation is 9.12. The calculation resulted in 21 patients per group. To compensate for 10% attrition rate, we included 24 patients per group. The total sample size is 96 patients” (Sample size calculation and statistical analysis, paragraph 2).

**The perioperative analgesic use should be more accurately described:**

Was intraoperative analgesia obtained using morphine? (Methods, paragraph 6). If yes, the total dose should be mentioned in Table 2 (I suppose the 10-15mg intraoperative morphine mentioned in table 2 is for analgesia, not for 6 hours anesthesia). Morphine was injected before the PACU in some patients (table 2 and data file): can you describe how the physician decided to inject or not morphine at the end of the surgery?

**Response:** (Methods, paragraph 6) Intraoperative morphine 10 – 15 mg was not for post operate analgesia. It was obtained for an average of 6 hours of anesthesia. So, we will move intraoperative morphine (mg) in table 2 to table 1.

Response: (table 2 and data file) The wording of MO_dose_beforePCA in the shared data file is a loading dose before the administration of Patient Controlled Analgesia (PCA) at PACU. It was not administered morphine before admitting the PACU.

At the PACU, the patients were asked pain scores every 15 minutes using a numerical rating scale. If the pain score is greater than or equal to 4 points, the patients were injected with 2 mg of morphine every 10 minutes until the patients reported pain scores of less than 4. Then, the patients started to use the PCA device in the post-operative period.

**Methods, end of paragraph 4:** no patient received preoperative gabapentinoid or opiod or NSAID. In the shared data file, 39/96 patients had gabapentinoid, 11 had NSAID and several had tramadol. Please explain these contradictory data.  

**Response:**  We wrote unclear. In the shared data file, the wording of Preop_Med is mean that current medications of patients. They were not premedication before day surgery. No patients received NSAIDs, gabapentinoid, or opioids on the morning of surgery. We change the word to “Current_Med” in the shared data file.

**Data analysis:**

**Results, first paragraph**: 10 of 96 patients were excluded because operative time < 4 hours - Why did you choose such a limit? This is quite in opposition with the “intention to treat analysis” mentioned in the last paragraph of methods. Please explain why we find 39 patients (and not 10) with an operative time < 240 min in the shared data file. Please clearly identify in the shared file which patients have been excluded.

**Response:**  We have incorrectly written. Ten patients were unable to use patient-controlled analgesia (PCA) device postoperatively that cause patients to drop out of clinical trials. We change the sentence to “There were three patients in the placebo-placebo group, two patients in nefopam-placebo group, two patients in placebo-nefopam group and three patients in nefopam-nefopam group were excluded from analysis. Because 10 patients were unable to use patient-controlled analgesia device postoperatively” (Results, paragraph 1). The sample size was anticipated by 10% attrition rates.

            Patients have been excluded who were number 31, 62 and 83 in placebo-placebo group, number 24 and 75 in nefopam-placebo group, number 69 and 73 in placebo-nefopam group, number 41, 66 and 72 in nefopam-nefopam group. Ten patients in the shared data file are highlighted in yellow (Shared data file). We were correcting code for each group (Shared data file, Group).

Response: The sentence “The main analysis was analyzed by intention-to-treat (ITT)” was deleted. And, we change the sentence to “Safety data analysis was analyzed by a statistical chi-square test” (Sample size calculation and statistical analysis, paragraph 2).

Response: We change the sentence to “Analysis of the Kruskal-Wallis test shows no significant difference in 24-hour postoperative morphine consumption between four groups, which were 18 [IQR 13.5-29], 20 [IQR 11-28.3], 17 [IQR 11.5-28.5], 13 [IQR 8.5-18.5] mg., respectively (p = 0.223)” (The results section in an abstract)

**How were the lacking data seen in the shared data file managed? (“NA”).**

**Response:** The lacking data (“NA”) were 10 patients who were unable to use patient-controlled analgesia (PCA) device postoperatively. Ten patients who dropped out of the study (Shared data file, highlighted in yellow).

**Results:**

Please replace in the third paragraph “similar” by “not different” (2 occurrences).

**Response: **We replace the word to “not different” in the third paragraph of results.

**Table 3:** are we dealing with nb of patients, nb of side effect occurrence? Can you mention cumulative data for patients (nb of patients having at least one occurrence of the side effect during the first 24 hours)?

**Response:**  The data were rechecked. We intended to report the occurrence of side effects. We change the word to “Variables, number of occurrences” in Table 3. We mentioned the number of patients having at least one occurrence of the side effect during the first 24 hours in the last row of table 3 (Table 3).

**I don’t understand Figure 2:** can you explain what each “time 0” is?

**Response:**  Figure 2 – Heart rate at time 0 is heart rate before starting the study drug before surgical incision (Figure 2, left).

Heart rate at time 0 is heart rate at the end of surgery (Figure 2, right).

# Peer review: Reviewer response for version 1

**DOI:** 10.5256/f1000research.25291.r65114

**Published:** 2020-07-07

**Authors:** Rattaphol Seangrung

**Affiliations:** 1 Department of Anesthesiology, Faculty of Medicine Ramathibodi Hospital, Mahidol University, Bangkok, Thailand

This study is a randomized, double-blinded, placebo-controlled trial that used nefopam 30 mg perioperative period for assessing its efficacy in spine surgery. The primary efficacy was defined as the percentage of a reduction in 24-hour morphine consumption during each time of nefopam administration (every four hours). However, one question I have is why the results of morphine consumption (table 2) were showed in the only milligram of morphine consumption at each time point.

Is the work clearly and accurately presented and does it cite the current literature?

Yes

If applicable, is the statistical analysis and its interpretation appropriate?

Yes

Are all the source data underlying the results available to ensure full reproducibility?

Yes

Is the study design appropriate and is the work technically sound?

Yes

Are the conclusions drawn adequately supported by the results?

Yes

Are sufficient details of methods and analysis provided to allow replication by others?

Yes

Reviewer Expertise:

pain management

I confirm that I have read this submission and believe that I have an appropriate level of expertise to confirm that it is of an acceptable scientific standard.

# Response

**Published:** 2020-07-28

**Authors:** Jatuporn Eiamcharoenwit

**Affiliations:** Department of Anesthesiology, Prasat Neurological Institute, Thailand

Thank you for your review and comment. Because 24 hours postoperatively total opioid consumption in the placebo group is less than in nefopam groups, and the result shows no significant difference in 24-hour postoperative morphine consumption between four groups. So, the primary outcome was to determine the milligram of 24-hour morphine consumption.

# Response

**Published:** 2020-08-13

**Authors:** Jatuporn Eiamcharoenwit

**Affiliations:** Department of Anesthesiology, Prasat Neurological Institute, Thailand

Dear Dr. Rattaphol Seangrung,

Thank you for giving a chance to improve our research article.

We changed the primary efficacy to “the primary efficacy was defined as the cumulative morphine dose received 24 hours postoperatively by Patient Controlled Analgesia (PCA) during each time of nefopam administration”

We already edited as new version. Hopefully, you would appreciate our revised version.

                                                                                                           

Regards,

Jatuporn Eiamcharoenwit
